# RNA sequence analysis data of *Peronospora destructor* maintained on onions

**DOI:** 10.1016/j.dib.2018.12.039

**Published:** 2018-12-18

**Authors:** Kazuki Fujiwara, Takashi Fujikawa, Akira Kawakami, Ryoichi Sonoda, Atsushi Miyasaka

**Affiliations:** a Kyushu Okinawa Agricultural Research Center, National Agriculture and Food Research Organization (NARO), Suya 2421, Koshi, Kumamoto 861-1192, Japan; b Institute of Fruit Tree and Tea Science, NARO, Fujimoto 2-1, Tsukuba, Ibaraki 305-8605, Japan

## Abstract

*Peronospora destructor* (Berk.) is an important biotrophic oomycete that causes downy mildew on onion (*Allium Cepa* L.) worldwide, especially in humid and temperate regions. The disease attacks bulb and seed production of onion, resulting in losses in yield and quality of bulbs. Epidemiological studies have increased our understanding and control of downy mildew on onion; however, little is known about the molecular aspects of *P. destructor* behavior during infection. Here, we isolated RNA from four samples of sporangia and sporangiophores of *P. destructor*, which were maintained by spore inoculation onto onions in a growth chamber. We then used an Ion PGM next generation sequencer to acquire and assemble the RNA sequences of *P. destructor*. By transcriptome shotgun assembly, we obtained 2335 contigs (N50, 884 nucleotides (nt); mean length, 881.6 nt). The data are accessible at NCBI (BioProject PRJNA391849). Our data resource will facilitate further studies of the molecular events during *P. destructor* infection.

**Specifications table**

**Table:** 

Subject area	*Agriculture, phytopathology*
More specific subject area	*Oomycete*
Type of data	*Table, text file*
How data was acquired	*RNA sequence data was obtained with an Ion PGM next generation sequencer*
Data format	*Raw reads in fasta format*
Experimental factors	*Sporangia and sporangiophore formation of* Peronospora destructor
Experimental features	Peronospora destructor *was maintained by spore inoculation onto onion shoots in a growth chamber, and the sporangia and sporangiophores were sampled for RNA extraction and RNA sequencing.*
Data source location	*Samples were collected from Yuni-cho, Hokkaido, Japan*
Data accessibility	Raw sequencing data are in the NCBI public repository, under Sequence Read Archive Accession Number SRR5755436, SRR5755437, SRR5755438, and SRR5755439 under the BioProject number PRJNA391849 (https://www.ncbi.nlm.nih.gov/bioproject/PRJNA391849).

**Value of the data**

• This dataset represents RNA sequences from sporangia and sporangiophore formation *of P. destructor.*
• This dataset will provide information about the molecular events occurring during *P. destructor* infection.
• The dataset presented here provide reference sequence data for further genetic studies and will be useful for comparative studies among *Peronospora* species.

## 1 Data

This article reports RNA sequence data from four samples of sporangia and sporangiophores of *P. destructor* maintained by spore inoculation onto onion shoots in a growth chamber. The raw data were deposited into the NCBI׳s Sequence Read Archive (SRA) (SRR5755436, SRR5755437, SRR5755438, SRR5755439) under the BioProject PRJNA391849. The RNA sequencing data are summarized in Table 1. Of the 2335 contigs, 2254 were identified as unique sequences from the *P. destructor* transcriptome because they were highly homologous to the known sequences of other related oomycetes (Supplemental file 1), and 81 were judged to be contaminants (Supplemental file 2).

**Table 1:** Summary of RNA sequencing of *Peronospora destructor* H-YUNI 1.

Total no. of reads obtained	3,018,850
Total no. of nucleotides obtained	418,316,149
Mean length of reads (nucleotides)	138.6
No. of assembled reads	1,992,197
No. of nucleotides in assembled reads	285,126,432
Assembly GC content (%)	50.2
No. of contigs	2335
No. of nucleotides in contigs	2,058,518
Contig size range (nucleotides)	436–4130
Contig N50^a^ (nucleotides)	884
Mean contig length (nucleotides)	881.6

a In the sum of generated contigs.

## 2 Experimental design, materials, and methods

### 2.1 Sample material

Onion seedlings with disease appearance of downy mildew were collected from Yuni-cho, Hokkaido in Japan on 7 July 2016. Sporangia of *P. destructor* on the surface of the leaves were gently collected into 0.0002% Tween 20 solution by using a soft brush and then inoculated onto onion seedlings, as described below.

Healthy onion bulbs were planted in a nursery and cultivated for a month under greenhouse conditions at daytime and nighttime temperatures of 25 °C and 18 °C, respectively. The onion seedlings developed from the bulbs were then spray inoculated with the collected spores described above. Sporangia production was carried out by the following three steps: First, the inoculated seedlings were moved to a KCSLPH-1400CT incubation box (Nippon Medical & Chemical Instruments Co., Ltd) and incubated in the dark for 12 h at 17 °C under highly humid conditions (100% relative humidity), as previously described [1]. Next, the incubated seedlings were moved to a LH-411S growth chamber (Nippon Medical & Chemical Instruments Co., Ltd, Osaka, Japan) and cultivated for two weeks under the following daily conditions: light for 18 h at 25 °C and dark for 12 h at 18 °C. Lastly, the cultivated seedlings were moved back to the incubation box and incubated under the same condition described above. When sporangia formed on the surface of the onion leaves, they were gently collected into sterilized distilled water with a soft brush and spray inoculated onto a new set of month-old onion seedlings. Cultivation was performed under the same conditions as described above. The spray inoculation and cultivation were performed more than three times, and then newly developed sporangia were collected into sterilized distilled water (approximately 1 × 10^6^ cells/ml) as *P. destructor* H-YUNI1 isolate. Four samples of sporangia suspension were prepared and used for separate RNA extractions.

### 2.2 RNA extraction and RNA sequencing

Total RNA was extracted from each of the four sporangia samples by using an RNeasy Plant Mini Kit (QIAGEN, Hilden, Germany). RNA templates were prepared with an Ion Total RNA-Seq Kit v2 (Thermo Fisher Scientific Inc., Waltham, MA, USA) and an Ion PGM Hi-Q OT2 Kit on an Ion OneTouch 2 System (Thermo Fisher Scientific), and then sequenced using an Ion PGM Hi-Q Sequencing Kit and a 318 Chip Kit v2 on an Ion PGM System next generation sequencer (Thermo Fisher Scientific), according to all the manufactures׳ recommendations. Assembly of sequence data and analysis were carried out using CLC Genomic Workbench software (CLC Bio, QIAGEN). For quality control, raw reads with a Phread quality score less than 20 and adaptors were removed. RNA contigs were assembled *de novo* by transcriptome shotgun assembly (word size = 21, bubble size = 138, contig length ≥ 500). Because no genome sequence data of *P. destructor* is available in public databases, the RNA contigs were subjected to NCBI BLAST analysis and contaminants including contigs annotated with regard to plant and bacteria were manually removed.

## Footnotes

Appendix A Supplementary data associated with this article can be found in the online version at https://doi.org/10.1016/j.dib.2018.12.039.
