# Supplementary material for: RNA sequence analysis data of Peronospora destructor maintained on onions
Source: Data Brief. 2018 Dec 18;22:693–6. doi: 10.1016/j.dib.2018.12.039 (PMC6329361; doi:10.1016/j.dib.2018.12.039)
Supplement: Supplementary file 1 — Supplementary material [file mmc1.docx]

Conflict of interest declaration and author agreement form

# RNA sequence analysis of *Peronospora destructor* maintained on onions

Kazuki Fujiwara^a^, Takashi Fujikawa^b^, Akira Kawakami^a^, Ryoichi Sonoda^a^, and Atsushi Miyasaka^a^

a Kyushu Okinawa Agricultural Research Center, National Agriculture and Food Research Organization (NARO), Suya 2421, Koshi, Kumamoto, 861-1192, Japan

b Institute of Fruit Tree and Tea Science, NARO, Fujimoto 2-1, Tsukuba, Ibaraki 305-8605, Japan

**Conflict of interest declaration**

We have no conflict of interest to declare.

**Author agreement**

This statement is to certify that all authors have seen and approved the being submitted. We warrant that the article is the author’s original work. We warrant that the article has not received prior publication and is not under consideration for publication elsewhere. On behalf of all co-authors, the corresponding author shall bear full responsibility for the submission.
